# Factors That Promote Vulnerability Versus Resilience Among Older Disaster Survivors

**DOI:** 10.1093/geroni/igaa057.1978

**Published:** 2020-12-16

**Authors:** Judith Robertson Phillips, Rachel Pruchno

## Abstract

Catastrophic environmental events, such as floods and hurricanes, are associated with widespread destruction and loss of life. Older adults, who often have health challenges and medical co-morbidities, appear to be at greater risk for adverse post-disaster outcomes, such as depression, worry, and medical uncertainty, than younger adults impacted by the same disaster. Researchers, therefore, are interested in identifying factors that tend to bring about vulnerability and adverse outcomes for older adults during and after a disaster, as well as factors that may generate resilience and psychological well-being for older adults. The purpose of this symposium is to present research based on multiple disaster experiences and to examine factors associated with vulnerability and resilience. The first two speakers introduce empirical findings from research that addresses flooding, the 2016 Baton Rouge flooding and the frequent flooding of coastal Louisiana brought about by coastal erosion. The next two presenters explore successful VA and Non-VA Home-Based programs during and after the 2017 Hurricane Maria in Puerto Rico and during the 2017 Atlantic hurricane season. The final speaker highlights the impact of lifetime trauma on the recovery of older adults following a natural disaster. Collectively, these presenters will provide evidence of how lifetime adversity is a factor promoting vulnerability for older adults after a disaster. Critically, they will also examine how age, disaster preparedness, intense patient tracking, VA support networks, and community resources and programs are protective factors generating resilience post-disaster for older adult populations.

